# Management of obesity in advanced chronic liver disease

**DOI:** 10.1016/j.jhepr.2026.101749

**Published:** 2026-01-30

**Authors:** Cyrielle Caussy, Sven M. Francque

**Affiliations:** 1 Hospices Civils de Lyon, Département Endocrinologie, Diabète et Nutrition, Hôpital Lyon Sud, 69495 Pierre-Bénite, France; 2 Univ Lyon, CarMen Laboratory, INSERM, INRA, INSA Lyon, Université Claude Bernard Lyon 1, 69495 Pierre-Bénite, France; 3 Department of Gastroenterology and Hepatology, Antwerp University Hospital, Antwerp, Belgium; 4 InflaMed Centre of Excellence, Laboratory of Experimental Medicine and Paediatrics, University of Antwerp, Antwerp, Belgium

**Keywords:** MASLD, bariatric surgery, anti-obesity medication, GLP-1-RA, fibrosis, lifestyle modification

## Abstract

Obesity has emerged as a major global health challenge and is closely associated with metabolic dysfunction-associated steatotic liver disease (MASLD) and metabolic dysfunction- and alcohol-associated liver disease (MetALD). Overall, an increased prevalence of obesity has been reported in patients with advanced chronic liver disease (ACLD), in whom it adversely impacts prognosis. Importantly, increased BMI is recognised as a modifiable risk factor for progression of liver disease, regardless of aetiology. The recent approval of effective and well-tolerated anti-obesity medications offers new opportunities, alongside therapeutic lifestyle changes and bariatric surgery, for the personalised management of patients living with obesity and associated liver disease.

## Graphical abstract

**Figure:**
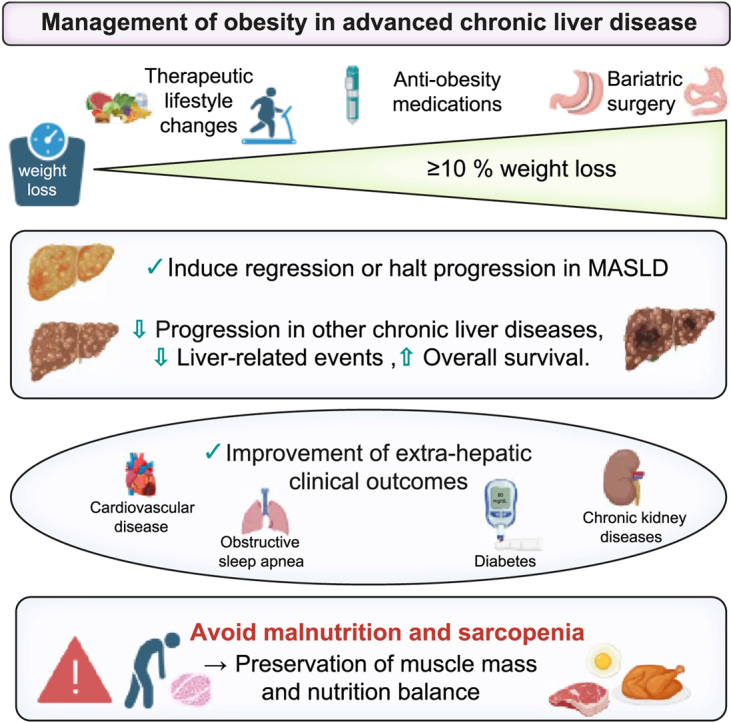

**Keypoints**

• The prevalence of obesity is increasing and it is frequently associated with advanced chronic liver disease (ACLD).
• Obesity is an independent risk factor for adverse clinical outcomes in chronic liver disease of several aetiologies, and a key cause of metabolic dysfunction-associated steatotic liver disease (MASLD).
• Weight-loss management in ACLD aims to induce regression or halt progression of MASLD, reduce obesity’s contribution to disease progression in other chronic liver diseases, lower liver-related risk, and improve overall survival.
• Therapeutic lifestyle changes alone have modest impact on weight loss, liver histology, and clinical outcomes.
• Novel anti-obesity medications can achieve ≥10% body weight loss, leading to meaningful histological improvement and reduced liver-related and cardiometabolic risks.
• Bariatric surgery is an effective and durable weight-loss intervention with associated liver and cardiometabolic benefits, though data are limited in advanced liver disease.

## Introduction

Obesity has emerged as a major global health challenge, with its prevalence reaching epidemic proportions across diverse populations, affecting an estimated 40% of the adult population in the US^1^ and 23% in Europe, according to the World Health Organization, which has recognised obesity as a chronic non-communicable disease.^2^^,^^3^ This rising epidemic of obesity is closely linked to a spectrum of metabolic and systemic complications including metabolic dysfunction-associated steatotic liver disease (MASLD) and metabolic dysfunction-associated steatohepatitis (MASH) and, in association with moderate alcohol consumption, metabolic dysfunction- and alcohol-associated liver disease (MetALD).^4^ MASLD is the most frequent cause of steatotic liver disease (SLD) with a global estimated prevalence of 30% ^5^ and MASLD-related decompensated cirrhosis and hepatocellular carcinoma (HCC) have become the primary indications for liver transplantation in many countries.^6^

However, the hepatic relevance of obesity is not limited to MASLD. Overall, an increased prevalence of obesity is reported in patients with various aetiologies of advanced chronic liver disease (ACLD) and impacts the prognosis of the disease, as an increase in BMI is recognised as a modifiable risk factor for progression of liver disease regardless of aetiology.^7^ Indeed, obesity is an independent risk factor associated with adverse clinical outcomes in patients with chronic liver disease, with approximately a three-fold higher risk of a first clinical decompensation event in patients with cirrhosis and obesity *vs*. patients with normal weight.^7^ In addition, class 3 obesity significantly increases the risk of acute-on-chronic liver failure and specifically renal failure in patients with decompensated cirrhosis.^8^ Sarcopenic obesity is associated with higher long-term mortality among patients with cirrhosis.^9^ The presence and number of features of the metabolic syndrome were also shown to impact the prognosis of patients with hepatitis B, illustrating that overweight and obesity should be assessed and managed in all patients with chronic liver disease,^10^ regardless of aetiology, in order to provide optimal care of all liver disease drivers.

Adding to this evolving landscape, a novel definition of obesity, classically based solely on BMI, has been proposed by a consensus of experts within the Lancet Commission. This novel approach reinforces the need to define clinical obesity as a chronic disease linked to the excess of adipose tissue and its impact on the function of organs and tissues, including the liver.^11^

Furthermore, the recent approval of novel, efficient and well-tolerated anti-obesity medications (AOMs) offers new opportunities for the personalised management of patients living with obesity and associated liver disease.

This review aims to synthesise the current evidence on the impact of different body weight loss management strategies currently available, including therapeutic lifestyle interventions, AOMs and bariatric surgery (BS). We focus on available data from patients with more severe liver disease subtypes, specifically MASH with fibrosis – particularly advanced fibrosis (stage F3) and compensated or decompensated ACLD/cirrhosis. In addition, this review summarises the current evidence on the long-term impact of these interventions on liver-related outcomes, as well as other relevant clinical outcomes associated with MASLD, such as cardiovascular (CV) and renal outcomes.

## Pathophysiological mechanisms

Although a detailed review of the mechanisms linking obesity to liver disease is beyond the scope of this review, a brief overview is provided to highlight the potential benefits of obesity management in chronic liver diseases.

In this regard, the new concept of clinical obesity is relevant, as it is not obesity *per se*, but rather dysfunctional adiposity, that is a major driver of liver disease in MASLD.[12], [13], [14]

Chronic inflammation and ischaemia of the (mainly but not exclusively) visceral adipose tissue leads to increased adipose tissue lipolysis and consequently to the release of free fatty acids that reach the liver. The adipocytokine imbalance and the release of multiple inflammatory mediators from adipose tissue also affect the liver, impacting all relevant intrahepatic cell types.^12^^,^^15^ This triggers a complex set of pathways in the liver, with vascular alterations,^16^ lipotoxicity, mitochondrial dysfunction, inflammatory pathways and direct activation of profibrogenic mechanisms ultimately leading to progressive fibrosis and triggering carcinogenesis.

Many factors beyond adipose tissue dysfunction contribute to the pathogenesis of MASLD/MASH, with substantial heterogeneity likely in the relative contribution of individual disease drivers across patients. Nevertheless, dysfunctional adiposity is regarded as one of the dominant drivers, even among the majority of individuals with MASLD and normal body weight.^17^ It is also important to emphasise that the liver is equipped with strong defence and repair mechanisms, adding another level of heterogeneity to the patient population and explaining why the liver phenotype might substantially vary despite comparable degrees of metabolic dysfunction.^17^

The liver’s capacity for repair and regeneration can lead to substantial disease regression, and even cirrhosis reversal, as observed in chronic liver diseases such as viral hepatitis. All this implies that, even in the absence of direct intrahepatic effects, interventions that substantially improve the metabolic drivers of MASLD/MASH might result not only in halting disease progression, but also in regression of the liver phenotype, particularly fibrosis,^18^ the most important histological prognostic feature of MASLD.

## Goals of weight loss intervention

The overall goal of weight loss is to improve or prevent obesity-related comorbidities. Thus, weight loss interventions are aimed at improving overall health, reducing the risk of adverse clinical outcomes, and improving quality of life.

It is widely acknowledged that achieving 5% weight loss is associated with meaningful clinical improvement in comorbidities and further weight loss greater than 10% is recommended in order to significantly improve clinical outcomes along with steatosis, liver inflammation and fibrosis.^19^ As the ultimate goal is to improve outcomes, it is important to recognise that management endpoints do not necessarily require reversal of disease severity to a less severe or normal state, as is often expected in MASH clinical trial definitions. Depending on the disease entity, the ability to halt disease progression through weight loss – even without reversal – might already translate into meaningful clinical benefit in the long run. Hence, it is crucial to consider the substantial heterogeneity among patients living with obesity, including body composition, comorbidities (as briefly touched upon regarding the liver in the previous section), as well as therapeutic responses to anti-obesity interventions. In addition, the effect of weight loss may vary according to the comorbidity. Some conditions, such as remission of type 2 diabetes (T2D), show a near-linear relationship, with greater improvement as weight loss increases. Other comorbidities may exhibit a non-linear association, with little or no additional benefit once a certain threshold of weight loss is reached.^20^ Interestingly, real-world data in the general population show greater improvement in MASLD with higher categories of weight loss.^21^ Moreover, in a longitudinal cohort of individuals with MASLD followed over a median 29-month period, weight loss >5% was associated with a non-significant trend toward a lower risk of liver-related events.^22^

**Fig. 1:**
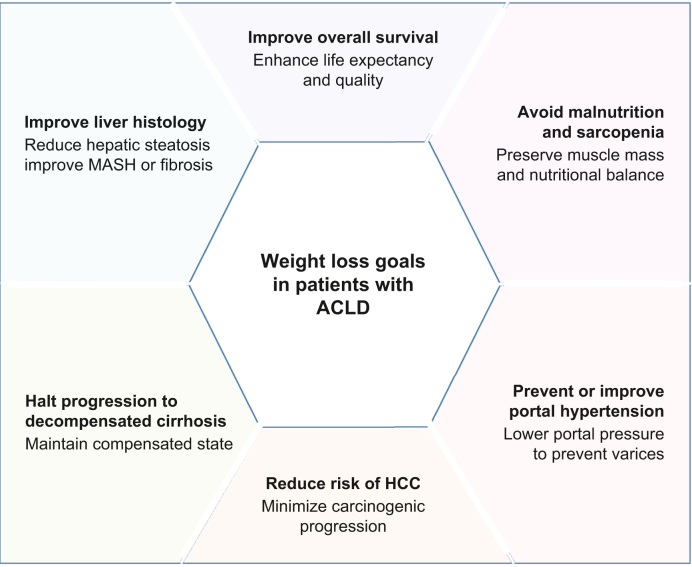
Weight loss goals in patients with ACLD. ACLD, advanced chronic liver disease; HCC, hepatocellular carcinoma.

In MASLD/MASH, efficacy is mainly assessed via liver biopsy.^23^ Regulatory frameworks and the EASL (European Association for the Study of the Liver) guidelines use two key endpoints: MASH resolution without fibrosis worsening and ≥1-stage fibrosis regression (0–4 scale) without MASH worsening for conditional/accelerated approval, considering that these endpoints are plausible (but not yet validated) predictors of clinical (liver) benefit.^24^ MASH resolution strongly predicts fibrosis regression,^25^ and fibrosis itself is the best histological prognostic marker.^26^^,^^27^ To date, although plausible, only one small cirrhosis study has linked fibrosis regression to reduced decompensation risk.^28^ Current endpoints emphasise lesion regression, although halting disease progression may also prevent adverse outcomes. Progression to cirrhosis is increasingly recognised as a valid clinical event in MASH trials.^29^ (Fig. 1)

**Fig. 2:**
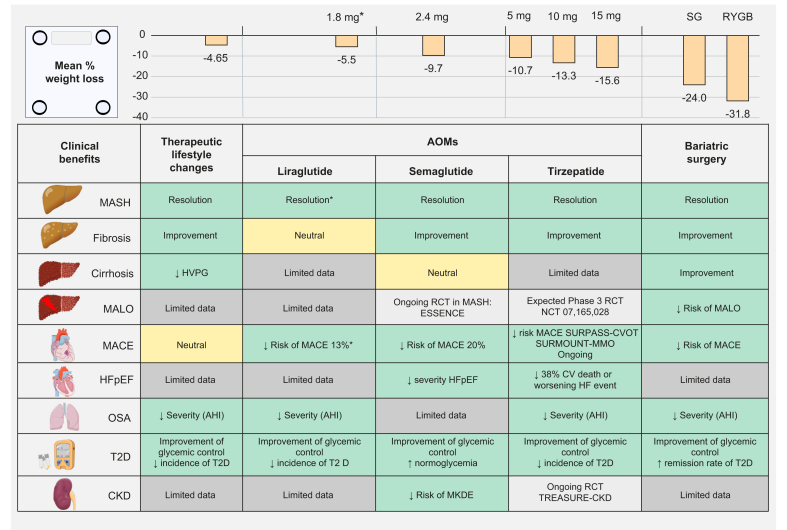
Summary of the effect of weight loss strategies on body weight and clinical outcomes. Mean percentage of weight loss is reported from studies available in patients with chronic liver disease, mainly MALSD (Table 1). The mean % change for semaglutide 2.4 mg corresponds to the mean of % WL calculated based upon reference^63^ and.^64^^∗^Lower dose than the AOM dose. AOM, anti-obesity medication; CKD, chronic kidney disease; CVD, cardiovascular disease; HFpEF, heart failure with preserved ejection fraction; KCCQ-CSS, Kansas City Cardiomyopathy Questionnaire clinical summary score; LVEF, left ventricular ejection fraction; MACE, major adverse cardiovascular events; MALOs, major adverse liver outcomes; MKDE, major kidney disease event; ; NA, not available; OSA, obstructive sleep apnoea; RYGB, Roux-en-Y gastric bypass; SG, sleeve gastrectomy; T2D, type 2 diabetes.

Focusing on cirrhosis and compensated ACLD (cACLD), key aspects of weight loss goals need consideration. These include improvement of liver histology, prevention or improvement of portal hypertension, prevention of the progression towards decompensated cirrhosis, avoidance of the development of malnutrition and sarcopenia and preservation of muscle functional capacity,^30^ reduction of the risk of HCC and improvement of overall survival (Fig. 2).

## Therapeutic lifestyle intervention

It is commonly acknowledged that weight loss achieved through lifestyle intervention, including hypocaloric diet and physical activity is a key element in the management of patients with obesity.^31^ Likewise, in patients with obesity and biopsy-proven MASH, weight loss through lifestyle intervention has demonstrated efficacy in improving hepatic steatosis, resolving MASH and promoting fibrosis regression.^32^ In addition, weight loss of ≥10% has been shown to improve features of MASLD, particularly steatosis and steatohepatitis.^32^^,^^33^ However, this threshold of 10% remains difficult to achieve through lifestyle intervention, even in randomised controlled trials (RCTs) with intensive coaching supported by dieticians and physical activity educators. In a prospective study performed in patients with biopsy-proven MASLD, less than 10% of patients achieved the optimal target of ≥10% weight loss^32^ (Table 1). In another study comparing lifestyle intervention *vs.* bariatric surgery, the lifestyle intervention arm achieved a mean weight loss of 5.5%, resulting in MASH resolution in 16% and a ≥1-stage fibrosis improvement in 23% of participants^34^ (Table 1). Furthermore, real-life data have shown the difficulties in adhering to lifestyle intervention: more than 20% of patients returned to baseline weight within 3 years, including 60% of them within 1 year.^35^ Of note, nutritional studies with lifestyle interventions performed in biopsy-proven cohorts are scarce, were performed mainly in patients with milder subtypes of MASLD and included only a small proportion of patients with fibrosis F3 (11-13%) and no patients with cirrhosis (Table 1).^32^^,^^34^

**Table 1:** Approved strategies for the treatment of obesity and their effect on MASH resolution and fibrosis improvement.

WL methods	Population	Study duration	Baseline BMI (kg/m^2^)	Stage of fibrosis severity	Average % of BWL/BMI change	MASH resolution	Fibrosis improvement	Study
**Therapeutic lifestyle changes**								
Hypocaloric diet combined with exercise	N = 293 Biopsy-proven MASH	52 weeks	31.3	F2: n = 59 (20%) F3: n = 31 (11%) F4: n = 0 (0%)	-3.8% BWL BMI: NA	25%	19 %	32
Lifestyle modification plus best medical care	N = 96 Biopsy-proven MASH	52 weeks	41.9	F2: n = 31 (38.8%) F3: n = 11 (13.8%) F4: n = 0 (0%)	-5.5% BWL -5.38% BMI	16%	23%	34

**Approved anti-obesity medications**								
Liraglutide *1.*8 mg*/day*∗	N = 27 Biopsy-proven MASH	48 weeks	34.2	F0–F2: n = 14 (54.0%) F3–F4: n = 12 (46.0%)	-5.5% BWL BMI: NA	39%	26.8% (ns)	52
Semaglutide 2.4 mg/week	N = 534 Biopsy-proven MASH F2 and F3	72 weeks	34.3	F2: n = 169 (31.6%) F3: n = 365 (68.4%) F4: n = 0 (0%)	-10.5% BWL BMI: NA	62.9%	36.8%	63
Semaglutide 2.4 mg/week	N = 47 MASH-compensated cirrhosis	48 weeks	34.6	F4: n = 47 (100%)	-8.8% BWL -9.0% BMI	34%	11%	64
Tirzepatide 5 mg/week	N = 47 Biopsy-proven MASH F2 and F3	52 weeks	36.1	F2: n = 17 (36%) F3: n = 30 (64%) F4: n = 0 (0%)	-10.7% BWL BMI: NA	44%	55%	118
Tirzepatide 10 mg/week	N = 47 Biopsy-proven MASH F2 and F3	52 weeks	36.6	F2: n = 257 (53%) F3: n = 22 (47%) F4: n = 0 (0%)	-13.3% BWL BMI: NA	56%	51%	118
Tirzepatide 15 mg/week	N = 48 Biopsy-proven MASH F2 and F3	52 weeks	35.9	F2: n = 22 (46%) F3: n = 26 (54%) F4: n = 0 (0%)	-15.6% BWL BMI: NA	62%	51%	118

**Bariatric surgery**								
Bypass and sleeve gastrectomy Gastric band Shunt	N = 109 Biopsy-proven MASH	52 weeks	48.9	F2: n = 32 (29.9%) F3: n = 26 (24.3%) F4: n = 5 (4.7%)	% BWL: NA -24.1% BMI	85.4%	46.3%	88
Bypass and sleeve gastrectomy Gastric band Shunt	N = 64 Biopsy-proven MASH	5 years	48.7	F2: n = 10 (15.6%) F3: n = 19 (29.7%) F4: n = 3 (4.7%)	% BWL: NA -26.0% BMI	84.4%	Fibrosis resolution∗: 50% F1–F2: 62.9% F3–F4: 45.5%	89
Bypass	N = 77 Biopsy-proven MASH	52 weeks	43.4	F2: n = 33 (42.8%) F3: n = 5 (6.5%) F4: n = 0 (0%)	-31.8% BWL -31.5% BMI	56%	37%	34
Sleeve gastrectomy	N = 79 Biopsy-proven MASH	52 weeks	40.8	F2: n = 28 (35.4%) F3: n = 9 (11.4%) F4: n = 0 (0%)	-24.0% BWL -23.91% BMI	57%	39%	34

BWL, body weight loss; MASH, metabolic dysfunction-associated steatohepatitis; NA, not available.

∗ Lower dose than the anti-obesity medication (liraglutide 3 mg daily); n.s., non-significant.

### Patients with cirrhosis

A few studies have reported the effects of a lifestyle intervention programme in patients with cirrhosis. A small retrospective study including patients with cirrhosis compared the impact of nutritional counselling by a multidisciplinary team *vs.* no dietary advice and reported a significantly higher survival rate among patients who received nutritional counselling by a multidisciplinary team.^36^ In another 6-week uncontrolled multicentre study, lifestyle intervention consisting of a hypocaloric diet and moderate exercise in patients with compensated cirrhosis (of a mixture of aetiologies, mainly viral hepatitis and alcohol-related), portal hypertension, and overweight or obesity, showed that weight loss was achievable and safe. The beneficial impact of the intervention on portal pressure invasively measured by hepatic venous pressure gradient (HVPG) was reported. Importantly, a significantly greater decrease in HVPG was observed in patients achieving ≥10% weight loss, and no clinical decompensation occurred.^37^ A more recent study including 27 patients with cirrhosis (again with a mix of aetiologies, only 15% were diagnosed with MASH) and portal hypertension, investigated the effects of a 12-week supervised exercise programme combined with a high-protein diet. Likewise, a significant reduction in HVPG was observed along with improvements in cerebral haemodynamics and cognitive function. Finally, the programme was well tolerated, with adherence exceeding 90%.^38^ Based upon these data, the ESPEN (European Society for Clinical Nutrition and Metabolism) guidelines recommend the implementation of lifestyle interventions aimed at promoting weight loss, particularly to reduce portal hypertension.^39^

### Other clinical outcomes

A few studies have assessed clinical outcomes associated with lifestyle intervention but not specifically in patients with ACLD (Table 2).^19^ The Look AHEAD study, including 5,145 patients with T2D and overweight, failed to demonstrate a significant reduction of major adverse CV events (MACE) with intensive lifestyle intervention compared to controls.^40^ However, the PREDIMED study, including 7,447 patients at high CV risk, demonstrated a lower incidence of MACE among participants assigned to a Mediterranean diet compared to those assigned to a low-fat diet; however, the study was not designed for weight loss management.^41^ Finally, studies have demonstrated efficacy of lifestyle interventions in improving and reducing the incidence of T2D in patients with overweight^42^ and improving the severity of obstructive sleep apnoea (Table 2).^43^

**Table 2:** Approved strategies for the treatment of obesity and their clinical outcomes.

Outcome	Therapeutic lifestyle changes	AOMs			Bariatric surgery
		Liraglutide	Semaglutide	Tirzepatide	
MALO	Limited data	Limited data	Ongoing RCT in MASH ESSENCE NCT04822181:^68^ n = 1,200 patients with biopsy-proven MASH F2 or F3	Limited data	SPLENDOR retrospective study: n = 168 patients with biopsy-proven MASH cirrhosis;^119^ n = 1,158 patients with biopsy-proven MASH without cirrhosis^104^
			Intervention: semaglutide 2.4 mg weekly *vs*. placebo		Intervention: bariatric surgery *vs*. matched non-surgical controls
			Duration: 240 weeks		Median follow-up: 7-10 years
			Effect: NA		Effect: ↓MALO MASH-cirrhosis: HR 0.28, 95% CI 0.12–0.64, *p =* 0.003 MASH without cirrhosis: aHR 0.12; 95% CI 0.02-0.63, *p* = 0.01
MACE	Look AHEAD study:^40^ n = 5,145 T2D and BMI ≥25 kg/m^2^	LEADER:^53^ n = 9,340 T2D and high CV risk	SELECT:^120^ n = 17,604 CVD and BMI ≥27 kg/m^2^	Ongoing RCT SURPASS-CVOT NCT04255433[82]: 13,299 patients with T2D, CVD and BMI ≥25 kg/m^2^ SURMOUNT–MMO NCT05556512,:^83^ ≈15,000 patient with CVD or multiple CV risk factors and BMI ≥27 kg/m^2^	SPLENDOR:^104^ n = 1,158 patients with biopsy-proven MASH without cirrhosis
	Intervention: intensive lifestyle intervention *vs*. diabetes support and education	Intervention: liraglutide 1.8 mg daily∗*vs*. placebo	Intervention: semaglutide 2.4 mg weekly *vs*. placebo	Intervention: SURPASS-CVOT: tirzepatide up to 15 mg weekly *vs*. dulaglutide 1.5 mg weekly SURMOUNT-MM0: tirzepatide up to 15 mg weekly *vs*. placebo	Intervention: bariatric surgery (RYGB, sleeve gastrectomy) *vs*. matched non-surgical controls
	Median follow-up: 9.6 years	Median follow-up: 3.8 years	Median follow-up: 39.8 months	Median follow-up: NA	Median follow-up: 7.0 years
	Effect: neutral HR 0.95; 95% CI 0.83- 1.09; *p =* 0.51	Effect: ↓13% MACE HR 0.87; 95% CI 0.78 to 0.97; *p* <0.001	Effect: ↓ 20% MACE HR 0.80; 95% CI 0.72 to 0.90; *p* <0.001	Effect: ↓ 8% MACE *vs*. dulaglutide HR 0.92; 95.3% CI 0.83 to 1.01	Effect: ↓ MACE aHR 0.30, 95% CI 0.12-0.72, *p* = 0.007
HFpEF	Limited data	Limited data	STEP-HF:^69^ n = 529 HFpEF and BMI ≥30 kg/m^2^	SUMMIT trial:^84^ n = 731 HFpEF and BMI ≥30 kg/m^2^	Limited data
			Intervention: semaglutide 2.4 mg weekly *vs*. placebo	Intervention: tirzepatide up to 15 mg weekly *vs*. placebo	
			Duration: 52 weeks	Duration: 104 weeks	
			Effect: ↓ severity HFpEF ↓KCCQ-CSS: 7.8 points; 95% CI 4.8 to 10.9; *p* <0.001	Effect: ↓ 38% CV death or worsening HF event HR 0.62; 95% CI 0.41 to 0.95; *p* = 0.026	
Obstructive sleep apnoea	Sleep AHEAD:^43^ n = 254 T2D and BMI ≥25 kg/m^2^ and OSA	SCALE Sleep apnoea:^56^ n = 359 with BMI ≥30 kg/m^2^	Limited data	SURMOUNT-OSA:^85^ n = 469 OSA and BMI ≥30 kg/m^2^	Systematic review and meta-analysis:^121^ n = 2,310 patients with OSA from 32 studies
	Intervention: intensive lifestyle intervention *vs*. diabetes support and education	Intervention: liraglutide 3.0 mg daily *vs.* placebo		Intervention: tirzepatide 10/15 mg *vs*. placebo	Intervention: bariatric surgery
	Median follow-up: 1 year	Median follow-up: 32 weeks		Median follow-up: 52 weeks	Median follow-up: NA
	Effect: ↓ severity of OSA ↓ in AHI: -9.7 events/h (95% CI -13.6 to -5.7; *p <*0.001)	Effect: ↓ severity of OSA: ↓ in AHI: -6.1 events/h (95% CI −11.0 to -1.2), *p =* 0.0150)		Effect: ↓ severity of OSA ↓ in AHI: −20.0 events/h (95% CI −25.8 to −14.2, *p <*0.001)	Effect: ↓ severity of OSA ↓ AHI (WMD = -19.3, 95% CI - 23.9 to -14.6) Rate of OSA remission: 65%; 95% CI 0.54 to 0.76
T2D	DPP study:^42^ n = 3,234 prediabetes and BMI >24 kg/m^2^ and glucose intolerance	SCALE Obesity and prediabetes:^49^ n = 2,254 prediabetes and BMI ≥27 kg/m^2^	STEP 10:^122^ n = 138 prediabetes and BMI ≥30 kg/m^2^	SURMOUNT-1:^123^ n = 2,539 prediabetes and BMI ≥30 kg/m^2^	SOS study:^124^ n = 603 patients with BMI ≥35 kg/m^2^ and T2D at baseline
	Intervention: intensive lifestyle intervention *vs*. standard lifestyle + metformin *vs*. standard lifestyle + placebo	Intervention: liraglutide 3.0 mg daily *vs*. placebo	Intervention: semaglutide 2.4 mg weekly *vs*. placebo	Intervention: tirzepatide 5 mg, 10 mg, or 15 mg or placebo	Intervention: bariatric surgery or conventional treatment
	Mean follow-up: 2.8 years	Duration: 160 weeks	Median follow-up: 52 weeks	Median follow-up: 176 weeks	Median follow-up: 10 years
	Effect: ↓ incidence of T2D ↓ 58%, 95% CI 48-66%.	Effect: ↓ incidence of T2D ↓ time to onset of T2D: HR: 0.21; 95% CI 0.13–0.34	Effect: ↑ proportion of patients achieving normoglycemia OR 19.8, 95% CI 8.7–45.2; *p<*0·0001	Effect: ↓ progression to T2D HR: 0.12; 95% CI 0.1 to 0.2; *p <*0.001	Effect: ↑ T2D remission OR: 6.3; 95% CI 2.1 to 18.9; *p* <0.001
CKD	Limited data	Limited data	FLOW:^71^ n = 3,533 T2D and CKD	TREASURE-CKD NCT05536804-ongoing: ≈150 patients with CKD and BMI ≥27 kg/m^2^	Limited data
			Intervention: semaglutide 1.0 mg weekly∗*vs*. placebo	Intervention: tirzepatide up to 15 mg *vs*. placebo	
			Median follow-up: 3.4 years	Median follow-up: NA	
			Effect: ↓ 24% of MKDE HR 0.76; 95% CI 0.66-0.88; *p =* 0.0003	Effect: NA	

AOM, anti-obesity medication; CKD, chronic kidney disease; CVD, Cardiovascular Disease; HFpEF, heart failure with preserved ejection fraction; KCCQ-CSS, Kansas City Cardiomyopathy Questionnaire clinical summary score; LVEF, left ventricular ejection fraction; MACE, major adverse cardiovascular events; MALO, major adverse liver outcomes; MKDE, major kidney disease event; NA, no data available; OSA, obstructive sleep apnoea; T2D, type 2 diabetes.

∗ Lower dose than the AOM dose.

## Anti-obesity medications (AOMs)

The different available AOMs can be categorised into a first generation and a more recent incretin-based generation. The first generation of AOMs mainly target appetite regulation in the brain, involving monoamine neurotransmitters such as norepinephrine, serotonin, and dopamine (phentermine-topiramate, naltrexone-bupropion). Additionally, another approved AOM induces malabsorption through the inhibition of gastrointestinal lipase (orlistat). This first generation of AOMs is limited by modest weight loss (generally <10%) and potential side effects or safety concerns that have limited their approval or reimbursement in some countries, as reviewed elsewhere.^44^^,^^45^

The new generation of AOMs encompasses the incretin-based therapies such as glucagon-like peptide-1 receptor agonists (GLP-1-RAs), which target the gut-brain axis. GLP-1-RAs increase satiety via the central nervous system, reduce gastric emptying and motility, and increase post-prandial insulin secretion in a glucose-dependent manner. These novel AOMs enable weight reductions of ≥5% with acceptable safety profiles and are also associated with significant improvements in several cardiometabolic outcomes, including cardiovascular and chronic liver diseases, particularly MASLD (Fig. 2, Table 2).

### Liraglutide 3 mg daily (Saxenda®)

The first GLP-1-RA approved as an AOM was liraglutide 3 mg daily (Saxenda®), which achieved the goal of 5% weight loss (in fact ranging from 6.0% to 8.0%) in the four RCTs from the SCALE program in patients with obesity.[46], [47], [48], [49]

#### Effects on liver features in patients with MASLD

Two studies have assessed the effect of liraglutide 3 mg daily *vs.* placebo in patients with MASLD. These studies are limited by their small sample sizes and relatively short durations (16-26 weeks), with the reduction in liver lipid content assessed using magnetic resonance imaging^50^ or ultrasound.^51^ Both studies demonstrated a significant reduction of hepatic steatosis induced by liraglutide. The LEAN study assessed the impact of liraglutide at a lower dosage (1.8 mg daily) in 26 patients with biopsy-proven MASH (including 46% of patients with advanced fibrosis) for 48 weeks *vs.* placebo. This landmark study demonstrated a significantly higher proportion of MASH resolution in participants treated with liraglutide (39%) *vs*. placebo (9%). However, there was no significant regression in liver fibrosis, even though a lower proportion of participants in the liraglutide group experienced progression of fibrosis *vs*. placebo^52^ (Table 1).

#### Other clinical outcomes

In patients with T2D and high CV risk, liraglutide 1.8 mg daily was associated with a significant 23% reduction in the risk of MACE.^53^ However, in two RCTs in patients with heart failure (HF), liraglutide 1.8 mg daily had a neutral effect on left ventricular systolic function^54^ and other composite endpoints, including time to death, time to rehospitalisation for HF, and time-averaged proportional change in N-terminal pro-B-type natriuretic peptide level from baseline to 180 days.^55^ Finally, liraglutide 3 mg daily has been shown to reduce obstructive sleep apnoea^56^ (Table 2).

### Semaglutide 2.4 mg weekly

The new generation GLP-1-RA semaglutide (Wegovy® 2.4 mg weekly) benefits from an enhanced resistance to proteolytic degradation by dipeptidyl peptidase 4 (DPP-4). This allows for a subcutaneous injection only once weekly and results in a higher magnitude of weight loss, with reductions of ≥10% (Fig. 2). In the STEP programme, which comprised randomised controlled trials evaluating the efficacy and safety of semaglutide 2.4 mg weekly *vs*. placebo in patients with a BMI ≥27 kg/m^2^ over 68 weeks,[57], [58], [59], [60] the mean percentage weight loss ranged from 9.6% up to 17.4%.[57], [58], [59], [60] A meta-analysis of pooled data from these RCTs reported a mean weight loss of -12.57% (95% CI -14.80 to -10.35) *vs.* placebo.^61^

#### Effects on liver features in patients with chronic liver disease

Phase II and III RCTs have investigated the effect of semaglutide in patients with MASH (Table 1). In a large phase II RCT evaluating semaglutide at doses different from those approved for obesity (0.1, 0.2, and 0.4 mg daily) in patients with MASH and fibrosis stages F2–F3, a higher proportion of MASH resolution and a significant reduction in hepatic steatosis were reported with semaglutide compared with placebo.^62^ However, semaglutide treatment did not significantly improve liver fibrosis after 18 months.^62^ Of note, a numerically lower proportion of participants treated with semaglutide had fibrosis progression compared with the placebo group.^62^ Several factors may explain this observation; however, consistent with other data we discuss in this review, it illustrates that MASH resolution achieved through improvement of the extrahepatic drivers of disease takes time to translate into fibrosis regression. The latter not only involves halting fibrosis progression but also reversing established fibrosis through fibrolytic mechanisms; thus, tissue repair processes must predominate over ongoing injury in the damage–repair balance to achieve fibrosis regression. MASH resolution would be expected to lead to fibrosis regression in the long run; however, small patient numbers, sampling variability and variability in treatment responses often limit the ability to demonstrate fibrosis regression, despite high rates of MASH resolution and, in this case, long treatment duration.

Recently, the interim analysis at 72 weeks of the ongoing phase III ESSENCE RCT, assessing semaglutide 2.4 mg once weekly in patients with MASH and fibrosis F2/F3, demonstrated significant improvement in both MASH resolution and liver fibrosis regression of ≥1 stage, leading to FDA conditional approval for the treatment of patients with fibrotic MASH.^63^ The fact that the phase III RCT achieved the fibrosis regression endpoint is in line with expectations based on the robust effect on MASH resolution, illustrating the importance of a sufficiently large sample size to overcome sources of variability when the effect size is modest.

Lastly, the efficacy and safety of semaglutide 2.4 mg weekly was assessed in a phase II RCT including patients with compensated MASH cirrhosis for 48 weeks.^64^ Among participants treated with semaglutide, MASH resolution was achieved in 34% and fibrosis improvement was observed in 11%, though the latter did not reach statistical significance *vs.* placebo (Table 1).^64^ In addition to semi-quantitative histological scoring by the pathologist, neither automated collagen quantification nor liver stiffness measurements showed significant improvement with semaglutide compared with placebo, supporting the conclusion that in patients with cirrhosis, despite weight loss and MASH resolution, a 1-year treatment duration does not confer fibrosis benefit. HVPG was not assessed; hence, a potential benefit on portal pressure, which could be hypothesised based on the aforementioned studies of lifestyle interventions in patients with cirrhosis of mixed aetiology, could not be assessed. However, the safety profile of semaglutide in this study population was satisfactory, with no hepatic or renal adverse events. Whether longer exposure to semaglutide 2.4 mg weekly and subsequent longer exposure to weight loss could result in a significant improvement of fibrosis or reduction in liver-related events in patients with compensated cirrhosis would require dedicated studies.

The incidence of liver-related events in patients treated with GLP-1-RAs has been studied in large health insurance system databases, mainly in patients with T2D treated with lower doses than 2.4 mg weekly. Interestingly, these retrospective studies underline a significant reduction in the risk of liver-related outcomes, including incidence of HCC, liver transplantation and hepatic decompensation in patients with MASH-cirrhosis treated with GLP-1-RAs *vs.* non-GLP-1-RA therapies.^65^ A lower risk of progression to cirrhosis was also reported in patients treated with GLP-1-RAs *vs*. DPP-4 inhibitors.^66^ However, in another retrospective population-based study from the UK, GLP-1-RAs were not associated with a lower incidence of cirrhosis compared with DPP-4 inhibitors.^67^ Hence, the hepatic benefit of GLP-1-RAs at anti-obesity doses still needs to be validated in dedicated RCTs. Indeed, the second part of the ongoing phase III ESSENCE RCT will provide important information on the efficacy of semaglutide to reduce progression to cirrhosis and liver-related events.^68^

#### Other clinical outcomes

Several RCTs have reported significantly lower rates of major CV events, HF-related clinical deterioration, and progressive chronic kidney disease (CKD) across diverse high-risk populations including patients living with obesity or T2D, positioning semaglutide as a therapy with a global cardio-metabolic health benefit (Table 2). In a large prospective RCT enrolling over 17,500 participants with BMI ≥27 kg/m^2^ without T2D and with established CV disease, the SELECT trial demonstrated a significant reduction of MACE. Over a mean period of 39.8 months, first-event MACE occurred in 6.5% of semaglutide-treated participants *vs.* 8.0% with placebo, yielding a significant 20% relative risk reduction. In another trial including 529 participants with obesity-related HF with preserved ejection fraction, semaglutide 2.4 mg weekly was associated with a 7.8-point greater improvement in exercise capacity and health status *vs.* placebo.^69^ In addition, a lower event rate of HF-related hospitalisation or urgent HF visits was observed even though the trial was not powered for clinical events. Finally, semaglutide 1 mg weekly was assessed in 3,533 participants with T2D and CKD in the FLOW RCT.^70^ The trial was stopped early for efficacy as the primary kidney composite endpoints including sustained ≥50% eGFR decline, kidney failure, or kidney-/CV-related death, occurred in only 18.7% *vs.* 23.2% of participants treated with semaglutide 1 mg weekly *vs*. placebo.^71^ Finally, emerging data suggest that GLP-1-RAs may reduce addictive behaviour and craving in adults with alcohol use disorder.^72^

### Tirzepatide 15 mg weekly

Tirzepatide, a dual GIP (glucose-dependent insulinotropic polypeptide) and GLP-1 receptor co-agonist, has been shown to induce significant weight loss, improve glycaemic control and insulin resistance, and reduce hepatic steatosis and both visceral and subcutaneous abdominal adipose tissue in patients with T2D and/or obesity. Several RCTs from the SURPASS programme in T2D^73^ and SURMOUNT programme in patients with BMI ≥27 kg/m^2^[74], [75], [76], [77], [78] led to its approval for the treatment of T2D (Mounjaro®) and obesity (Zepbound®). In patients with obesity, weight loss achieved at the highest effective dose (15 mg weekly) ranged from 14.7% to 20.9%, approaching the magnitude of weight loss observed with BS (Fig. 2, Table 1). Further confirmed by a meta-analysis of pooled data from six RCTs on individuals with overweight or obesity, tirzepatide induced a mean weight loss of −16.32% (95% CI -18.35 to -14.29, *p* <0.0001) *vs.* placebo when all doses were pooled.^79^ In addition, the proportion of participants who achieved a weight loss ≥20% ranged from 30.8% to 69.5%. A head-to-head comparison between semaglutide 2.4 mg and tirzepatide 15 mg weekly in 750 participants with BMI ≥27 kg/m^2^ for 72 weeks confirmed a higher weight loss with tirzepatide *vs.* semaglutide: -21.8 *vs.* -15.6 %, *p* <0.001.^74^ This greater weight loss was also reported in a retrospective analysis of a real-world healthcare database.^80^

#### Effects on liver features in patients with MASLD

In a phase II RCT including patients with MASH and fibrosis stages F2/F3, tirzepatide significantly increased the proportion of patients achieving MASH resolution without fibrosis worsening in a dose-dependent manner, with a placebo-subtracted effect of up to 52%. Additionally, a numerically higher proportion of participants had an improvement of ≥1stage of fibrosis without worsening of MASH, with a placebo-subtracted effect up to 25% but no clear dose-dependency. There are currently no studies in patients with MASH-cirrhosis (Table 1). A study assessing the pharmacokinetics and tolerability of tirzepatide in participants with hepatic impairment (up to Child-Pugh C, with or without T2D) compared with healthy participants with normal hepatic function reported a similar pharmacokinetic profile, indicating that no dose adjustment is required in cases of impaired liver function. However, long-term safety data in this population are lacking; therefore, the use of tirzepatide is not recommended in patients with decompensated cirrhosis and should be prescribed with caution in patients with compensated cirrhosis.^81^ Finally, the efficacy of tirzepatide in patients with MASH will need to be confirmed in a large phase III RCT assessing clinical outcomes, which is ongoing (NCT07165028).

#### Clinical outcomes

Recently, a large RCT assessing the impact of tirzepatide on CV outcomes in patients with T2D and CV disease (NCT04255433,^82^) reported an 8% reduction in MACE *vs*. dulaglutide, demonstrating non-inferiority compared with the known beneficial effect of dulaglutide. Another large RCT in patients with BMI ≥27 kg/m^2^ is ongoing (NCT05556512,^83^). Likewise, an RCT assessing the effect of tirzepatide on renal outcomes in patients with overweight or obesity and CKD is ongoing (NCT05536804). Notably, in an RCT including 731 participants with HF with preserved ejection fraction, tirzepatide up to 15 mg weekly led to a significantly lower risk of a composite of CV death or worsening HF *vs.* placebo.^84^ Likewise, in patients with obesity and obstructive sleep apnoea, tirzepatide significantly reduced the severity of obstructive sleep apnoea *vs.* placebo (Table 2).^85^

## Metabolic and bariatric surgery

### Histological improvement of MASH and fibrosis

BS, also referred to as metabolic surgery (MS), is the most effective intervention in terms of body weight reduction, leading to significant long-term weight loss concomitant with metabolic improvement. The effects of BS on features of MASLD/MASH in patients with obesity have been extensively studied in several retrospective or prospective cohort studies, demonstrating its efficacy in reducing or even completely reversing steatosis or MASH, and further confirmed in meta-analyses.^86^^,^^87^ However, the majority of the patients enrolled in these studies did not have advanced fibrosis and patients with decompensated cirrhosis are classically excluded, as decompensated cirrhosis is a contraindication to BS.

A 52-week multicentre open-label randomised trial in patients with biopsy-proven MASH comparing the efficacy of bariatric–metabolic surgery (either Roux-en-Y gastric bypass [RYGB] or sleeve gastrectomy [SG]) *vs.* lifestyle intervention demonstrated that BS is more effective than lifestyle intervention to achieve MASH resolution and fibrosis improvement of ≥1 stage.^34^ However, the trial included only 18.8% of patients with fibrosis stage F3 and no patients with F4 (cirrhosis) (Table 1).

Another prospective study in patients with biopsy-proven MASH with longitudinal follow-up and repeated liver biopsies at 1 and 5 years after BS showed that liver fibrosis improvement of ≥1 stage is observed in many patients at 1 year^88^ and further substantially improved after 5 years.^89^ The time course is relevant, as some metabolic effects occur early, as well as the resolution of MASH (already in 80% at 1 year), yet most of the cases of fibrosis regression were only observed at 5 years. This again illustrates the concept that improving the drivers of disease can lead to fibrosis regression; however, in the absence of direct intrahepatic effects on fibrogenesis, fibrosis regression induced by weight loss takes time to occur (particularly with the current definition of a ≥1 stage improvement on the semi-quantitative 0-4 fibrosis scale) (Table 1).

These data also highlight that sustained weight loss is important to achieve fibrosis improvement. Interestingly, 34.3% of participants had advanced fibrosis at baseline and even this group achieved fibrosis resolution at 5 years in 45.5% of cases (*vs.* 62.9% in participants with lower stages of fibrosis at baseline), showing the substantial long-term liver benefit of weight loss even in more advanced cases.^89^ Conversely, there were also non-responders in terms of fibrosis regression, despite substantial weight loss. Absence of response, or even worsening, was histologically associated with more persistent MASH, and metabolically with less weight loss and higher BMI at 5 years. In line with these results, another retrospective study including 66 participants with biopsy-proven advanced liver disease, including 36 patients with advanced fibrosis (stage F3–F4), reported that advanced fibrosis persisted in 47% after BS within a follow-up period of 5.6 years.^90^

Hence, based upon the current evidence, histological fibrosis improvement is observed in 45.5% to 53% of cases with MASLD-related advanced fibrosis and typically occurs beyond 1 year and up to 5 years after BS. This important information needs to be clearly delivered to patients with advanced fibrosis undergoing BS regarding the timing of expected improvement and potential risk of persistent advanced fibrosis. Moreover, long-term longitudinal data suggest that weight regain, defined as an increase of more than 20% from the postoperative nadir weight, occurs in approximately 53% of patients after RYGB and in up to 57% of patients after SG.^91^^,^^92^

### Type of surgical procedure and pre-operative assessment

The type of surgical procedure is also potentially important, especially in patients with more advanced liver disease. RYGB is usually associated with a higher proportion of body weight loss than SG and is associated with a higher proportion of metabolic improvement, such as T2D resolution.^93^ There is no formal head-to-head comparison of the efficacy of RYGB *vs.* SG in liver improvement available in patients with advanced fibrosis. The prospective study from Verrastro *et al.*, including mainly participants with fibrosis ≤F2, observed no significant difference between RYGB and SG in MASH resolution or fibrosis improvement.^34^ However, in a subgroup analysis focusing on the more severe participants (NAFLD activity score ≥4 [on a 0-8 scale] and fibrosis F2/F3), fibrosis improvement occurred more frequently in the RYGB group: 80% *vs.* 70% in the SG group.^34^ In line with these results, in a retrospective study, also focusing on patients with advanced liver disease (defined by fibrosis ≥F3 or SAF activity score A3/A4 [on a 0-4 scale]), older age and SG were the only independent predictors of persistent advanced fibrosis after adjustment for duration of follow-up.^90^

Even though SG may be less effective than RYGB, it is often preferred in patients with cirrhosis/cACLD as, in this specific population, it may be associated with lower mortality *vs*. RYGB according to a retrospective study.^94^ In addition, SG is technically a less complex surgical procedure, which also preserves the usual endoscopic access to the biliary tree. Finally, unlike RYGB, SG does not induce malabsorption and therefore may reduce the risk of malnutrition in patients with cirrhosis, who often have sarcopenia; this is a particular concern in MASH-cirrhosis, as obesity itself is frequently associated with sarcopenia.

Among individuals with class III obesity and cACLD, a subset may have clinically significant portal hypertension (CSPH), which needs to be assessed prior to BS. The concept of CSPH refers to the threshold of portal hypertension severity (*i.e.* a HVPG of ≥10 mmHg) associated with an increased risk of hepatic decompensation events and death.^95^ It was shown that a HVPG ≥16-20 mmHg is associated with a very high risk of death (44%) in patients with cirrhosis undergoing elective extrahepatic surgery.^96^ In the absence of dedicated studies for BS, the same principles used for other types of major abdominal surgery should be applied, with an HVPG ≥10 mmHg indicating high surgical risk.^97^ Besides these general rules, multidisciplinary expertise remains a key element in the decision to perform BS in patients with cirrhosis, with careful assessment of the benefit-risk ratio. In patients with cirrhosis and CSPH with an indication for BS, transjugular intrahepatic portosystemic shunt (TIPS) may be, on a case-by-case basis, an option to reduce portal hypertension in an attempt to de-risk the procedure or expand the options in terms of type of surgery. In a small retrospective study comparing patients with CSPH undergoing TIPS prior to surgery to a control group (almost all without CSPH and obviously no TIPS), TIPS placement was not only safe, but was also associated with similar outcomes after surgery as the control group, although the study is limited by its small sample size and potential lack of statistical power.^98^ This supports the concept that BS could be safe in patients who have previously undergone TIPS and that it may enable BS in patients with cirrhosis/ACLD and CSPH.

Finally, alcohol consumption needs to be carefully assessed, both as a baseline contributing factor to liver disease (leading to a diagnosis of mixed disease aetiologies such as MetALD) but also because BS is a risk factor for the development or aggravation of alcohol use disorder.^99^ Given the altered alcohol absorption after BS, this can lead to accelerated liver disease progression post-BS.

### Clinical outcomes

There is strong evidence supporting the durability of weight loss after BS, with studies demonstrating sustained weight loss for up to 20 years and an associated reduction in overall mortality..^100^^,^^101^ In addition, several studies have reported a high proportion of resolution or significant improvement in obesity-related comorbidities including T2D, dyslipidaemia, hypertension or obstructive sleep apnoea.^102^ In a meta-analysis using patient-level survival data reconstructed from prospective controlled trials and high-quality matched cohort studies of 174,772 participants, BS was associated with substantially lower all-cause mortality rates and longer life expectancy than non-surgical standard obesity management including mostly lifestyle interventions.^103^ In addition, there is recent evidence of a significant reduction of major adverse liver-related outcomes (MALOs) (*i.e*. progression to clinical or histological cirrhosis, decompensation events [such as portal hypertensive bleeding, ascites requiring treatment, hepatic encephalopathy], development of HCC, liver transplantation, or liver-related mortality) and MACE after BS in patients with biopsy-proven MASH *vs.* BMI-matched non-surgical controls.^104^ Interestingly, a long-term follow-up of a prospective cohort with biopsy-proven MASH and repeated liver biopsy at 1 year, not only confirmed the baseline prognostic value of MASH and fibrosis, but also demonstrated that histological remission of MASH or significant improvement of fibrosis from F2–F4 to F0–F1 were associated with significant improvement in overall survival after BS *vs*. those with persistent lesions^105^ (Table 2). The subgroups were too small for a more granular assessment of the causes of death, and beyond mortality, MALOs were not reported in this study. Nevertheless, the study provides further evidence that MASH is an important comorbidity within the spectrum of metabolic syndrome, contributing to disease burden, and highlights the multifaceted therapeutic potential of BS in this context.

## Emerging endoscopic procedures for obesity management

Endoscopic bariatric and metabolic interventions are gaining attention as potential alternatives to traditional BS.^106^ Techniques such as the intragastric balloon^107^ ^18^ and endoscopic sleeve gastroplasty^108^ have shown promising results in improving non-invasive biomarkers associated with MASLD, with some evidence suggesting histological benefits. However, a systematic review and meta-analysis that included only two studies on duodenal mucosal resurfacing found no significant reduction in liver lipid content as assessed by MRI-derived proton density fat fraction.^109^ Another meta-analysis examining the impact of endoscopic bariatric procedures on MASLD indicates potential improvements in hepatic steatosis, liver enzyme levels, and insulin sensitivity. Still, these findings require validation through larger, long-term prospective trials incorporating liver biopsies or, in the future, well-validated non-invasive tests with high diagnostic and/or prognostic accuracy.^110^ Notably, there are currently no published studies evaluating these procedures in patients with cACLD/cirrhosis.

## Gaps in knowledge and perspectives

In patients with advanced fibrosis and cACLD/cirrhosis, obesity management is crucial, offering potential histological improvement (notably in MASH) and reduction of MALOs. However, evidence in advanced fibrosis remains limited, and further dedicated studies are warranted.

The impact and safety of novel AOMs need to be further investigated in patients with cACLD/cirrhosis and obesity in order to demonstrate their efficacy on MALOs and their safety in patients with MASH and/or other concomitant causes of liver disease. These investigations will need to incorporate strategies to minimise muscle loss during the use of AOMs, including optimal protein intake and the physical activity recommendations detailed in,^30^ as well as body composition assessments to provide a comprehensive evaluation of the benefits and risks of AOM use in patients with cACLD/cirrhosis. In addition, direct head-to-head comparison of the benefit of BS and AOMs is lacking and will be crucial to further determine the most suitable strategy in patients with cACLD or cirrhosis.

In addition, a better understanding of the heterogeneity of the therapeutic response to AOMs is needed, as only about one-third of patients achieve ≥15% weight loss with GLP-1-RAs, while non-responders (or those who are intolerant) complicate real-world deployment. The patient profile, such as T2D status, may modify efficacy as RCTs performed in patients with T2D consistently report less weight loss *vs*. non-T2D participants. However, these differences in weight loss according to T2D status were not observed in *post hoc* analyses from three RCTs investigating the effect of semaglutide *vs.* placebo in adults with MASLD.^111^

The durability and sustainability of weight loss is another concern. Extension phases of SURMOUNT-4^77^ and STEP-1^112^ revealed rapid weight regain after treatment discontinuation, highlighting obesity’s chronic, relapsing nature and the need to maintain AOMs long term. In contrast, long-term data show that BS induces durable weight loss and consistent improvements in clinical outcomes. Hence, sequential or combination strategies using AOMs and BS may be necessary in some individuals depending on therapeutic responses.

Importantly, next-generation AOMs, such as dual GLP-1/glucagon agonists (survodutide) and triple GIP/GLP-1/glucagon agonists (retatrutide) have shown promising results in both obesity management^113^^,^^114^ and in MASLD/MASH^115^^,^^116^ in phase II RCTs. These therapies are currently under investigation in large phase III RCTs for the treatment of obesity (NCT05929066, NCT06066515) and MASH (NCT07165028, NCT06632444, NCT06632457). Interestingly, a potential enhanced liver benefit could be expected using these therapies as glucagon has a direct effect on the liver.^117^

Finally, cost-effectiveness analyses increasingly favour early pharmacotherapy in cirrhosis, but long-term modelling must incorporate drug cycling, monitoring burden, and potential downstream transplant savings. Although pharmacological and surgical options are expanding, they must be embedded within sustained lifestyle intervention and multidisciplinary management to address cardiometabolic comorbidities comprehensively and promote durable liver health.

## Abbreviations

ACLD, advanced chronic liver disease; AOM, anti-obesity medication; BS, bariatric surgery; cACLD, compensated advanced chronic liver disease; CKD, chronic kidney disease; CSPH, clinically significant portal hypertension; CV, cardiovascular; DPP-4, dipeptidyl peptidase 4; GIP, glucose-dependent insulinotropic polypeptide; GLP-1-RAs, glucagon-like peptide-1 receptor agonists; HCC, hepatocellular carcinoma; HF, heart failure; HVPG, hepatic venous pressure gradient; MACE, major adverse cardiovascular events; MALOs, major adverse liver outcomes; MASLD, metabolic dysfunction-associated steatotic liver disease; MASH, metabolic dysfunction-associated steatohepatitis; MetALD, metabolic dysfunction- and alcohol-associated liver disease; RCT, randomized controlled trial; RYGB, Roux-en Y gastric bypass; SG, sleeve gastrectomy; T2D, type 2 diabetes; TIPS, transjugular intrahepatic portosystemic shunt.

## Authors' contributions

CC drafting of the manuscript, SF critical revision of the manuscript, both authors approved the final version of the manuscript.

## Financial support

No financial support was received to produce this manuscript.

## Conflicts of interest

CC received consultant fees from Gilead, NovoNordisk, AstraZeneca, Lilly, E-scopics, MSD, Bayer, Corcept, Boeringher Ingelheim, E-scopics and Echosens, grant support from Gilead, NovoNordisk and Echosens. SF holds a senior clinical investigator fellowship from the Research Foundation Flanders (FWO) (1802154 N). His institution has received grants from Astellas, Falk Pharma, Genfit, Gilead Sciences, GlympsBio, Janssens Pharmaceutica, Inventiva, Merck Sharp & Dome, Pfizer, Roche. He has acted as consultant for Abbvie, Actelion, Aelin Therapeutics, AgomAb, Aligos Therapeutics, Allergan, Alnylam, Astellas, Astra Zeneca, Atheneum Partners GmbH, Bayer, Boehringer Ingelheim, Bristoll-Meyers Squibb, Byondis, CSL Behring, Coherus, Echosens, Dr. Falk Pharma, Eisai, Enyo, Galapagos, Galmed, Genetech, Genfit, Genflow Biosciences, Gilead Sciences, Intercept, Inventiva, Janssens Pharmaceutica, PRO.MED.CS Praha, Julius Clinical, Madrigal, Medimmune, Merck Sharp & Dome, Mursla Bio, NGM Bio, Novartis, Novo Nordisk, Promethera, Roche, Siemens Healthineers, V4Cure, Weatherden. SF has been lecturer for Abbvie, Allergan, Bayer, Eisai, Genfit, Gilead Sciences, Janssens Cilag, Intercept, Inventiva, Merck Sharp & Dome, Novo Nordisk, Promethera, Siemens.

Please refer to the accompanying ICMJE disclosure forms for further details.
